# Effect of a GnRH Antagonist on Anti-Müllerian Hormone and Follicular Population in Anestrous Bitches

**DOI:** 10.3390/ani13152511

**Published:** 2023-08-03

**Authors:** Cynthia Marchetti, Mariela Grisolia Romero, Marcelo Priotto, Marcela Faya, Cristina Gobello

**Affiliations:** 1 Center of Reproductive Physiology, Faculty of Veterinary Sciences, National University of La Plata, La Plata 1900, Argentina; cynthia_marchetti@hotmail.com (C.M.); mari_griso@hotmail.com (M.G.R.); 2 Consejo Nacional de Investigaciones Científicas y Técnicas, Buenos Aires 2290, Argentina; marcela_faya@yahoo.com.ar; 3 Small Animal Clinics, Faculty of Agricultural Sciences, Catholic University of Córdoba, Córdoba 5000, Argentina; priottomarcelo54@gmail.com

**Keywords:** canine, GnRH analog, Müllerian inhibitory substance, follicular reserve

## Abstract

**Simple Summary:**

This study was carried out to describe the effect of a new GnRH antagonist, acyline, on ovarian follicular numbers and the blood anti-Müllerian hormone (AMH) in non-cycling bitches. Acyline was serially administered for 60 days; then the females were followed up for an additional 45 days and spayed. Blood samples were taken and ovaries microscopically studied. None of the female dogs presented an estrus cycle during treatment. One bitch presented a normal cycle after it. The total number and proportions of ovarian follicles were normal. AMH diminished to very low values during treatment and rapidly increased after its effect. Acyline rapidly and reversibly prevented the initiation of cycling without affecting follicle count but diminishing AMH concentrations. AMH appears to be a functional biomarker to assess ovarian response to this inhibitory pharmacological protocol.

**Abstract:**

The objective of this study was to describe the effect of the third-generation GnRH antagonist, acyline, on ovarian follicular population and serum anti-Müllerian hormone (AMH) concentrations in female dogs. Four late anestrous bitches were administered 330 μg/kg SC acyline every 10 days for 60 days and followed up for 45 days. Blood samples were drawn on days −1, 15, 30, 45, 60, 75, 90 and 105 for AMH determination. Then, the females were ovariectomized and the excised ovaries were gross and histologically evaluated. The total ovarian follicles were counted. None of the female dogs presented estrus during treatment. Only one bitch presented an ovulatory estrus 20 days after treatment. The total number of ovarian follicles in these bitches was 96,200.10 ± 26,125.12, with 84.13%, 11.36%, 7.8% and 0.01% corresponding to primordial, primary, secondary and antral structures, respectively. Pretreatment AMH concentrations were 0.62 ± 0.17 ng/mL. This hormone varied throughout the study period (*p* < 0.01), diminishing to nadir values during treatment to then rapidly recover after its effect (0.2 ± 0.05 vs. 0.67 ± 0.22 ng/mL; *p* < 0.01). Acyline rapidly and reversibly prevented the initiation of cycling without affecting follicle count but diminishing serum AMH concentrations.

## 1. Introduction

Gonadotropin-releasing hormone (GnRH) antagonists competitively block and inhibit GnRH-induced GnRH receptor gene expression leading to an immediate, dose-dependent, pituitary suppression of the gonadal axis. In female dogs, third-generation GnRH antagonists prevented ovulation, interrupted mid-pregnancy, partially prevented GnRH agonist stimulation and resolved pyometras [1,2,3,4]. In male dogs, the third-generation GnRH antagonist acyline rapidly decreased gonadotropins (FSH and LH) and testosterone for 9 days after a single subcutaneous administration without any hematological, serum biochemical, local or systemic side effects [1,5]. Although the use of these new GnRH antagonists appears promising in domestic dog reproduction, there are still many aspects that remain to be unveiled. Suppression of ovarian folliculogenesis with GnRH antagonists may have potential not only in contraception but also in the prevention of chemotherapy infertility as well as in the improvement of estrus induction protocols [6,7,8].

Anti-Müllerian hormone (AMH) or Müllerian inhibitory substance is a glycoprotein belonging to the transforming growth factors (TGF-β) that, in mature females, is exclusively secreted by granulosa cells of primary, secondary and small antral follicles of the ovary [9]. Anti-Müllerian hormone also suppresses the initiation of primordial follicle growth and reduces the sensitivity of antral follicles to follicle-stimulating hormone [9]. This hormone is considered an indirect biomarker of the total number of ovarian follicles [10]. In bitches, AMH concentrations are known to increase in the last three weeks of anestrus up to six days before ovulation when it begins to decrease [11,12]. With the ovaries being the only source of this hormone, spaying markedly decreases AMH concentrations [13].

Information on the concentrations of AMH in GnRH-antagonist-treated bitches might be useful not only to monitor the suppression of the ovarian activity but also to predict and evidence the eventual resumption of gonadal function after the induced quiescence. In this study, late anestrus was selected to work in as it is a period of the canine estrus cycle in which folliculogenesis is activated [14,15]. The objective of this study was to describe, for the first time, the effect of the third-generation GnRH antagonist, acyline, on ovarian follicular population and serum AMH concentrations in late anestrous bitches.

## 2. Materials and Methods

### 2.1. Animals, Treatment Protocol and Follow-Up

Four 2.5 to 3.5 years, 8.4 to 9.8 kg, late anestrous beagle bitches (Bitch 1, 2, 3, 4) that belonged to the Catholic University of Cordoba, Argentina, were included in this study. Late anestrus was diagnosed by history (>5 months of previous estrus cycle), vaginal cytology (neither superficial nor large intermediate cells) and basal progesterone concentration [16]. These female dogs had been cycling regularly every 7 ± 1 months.

The females were administered 330 μg/kg SC acyline every 10 days for 60 days without any local side effects. Acyline was provided in a lyophilized powder that was suspended in sterile distilled water at a concentration of 2 mg/mL. The dosage was selected based on a previous report [5]. Then, the animals were followed up 45 days after treatment. This study was reviewed and approved by the Animal Care and Use Committee of the Catholic University of Córdoba, Argentina (18/002), and all experiments were conducted under the guidelines established in the Guide for the Care and Use of Laboratory Animals, USA.

### 2.2. Blood Sampling and Hormone Measurements

Blood samples were drawn by peripheral venepuncture before and after (days 15, 30, 45, 60, 75, 90, 105) the beginning of the treatment (day 1). Serum was obtained after centrifugation of the samples at 3200 rpm (600 g) for 15 min at 4 °C and stored frozen at −70 °C until AMH (Elecsys^®^, Cobas, Roche Diagnostics International Ltd., Rotkreuz, Switzerland) analysis. This assay was previously validated for dogs and confirmed by us [11]. The AMH electrochemiluminescence immunoassay was used according to the manufacturer instructions. The sensibility and the intraassay CV of the kit were 0.01 ng/mL and <5%, respectively. The maximum detection limit was 23 ng/mL.

### 2.3. Ovariectomies, and Gross Histological Evaluation of the Gonads

The females were ovariectomized immediately after last blood sample (day 105) by routine procedures [17]. The excised ovaries were measured (cm), weighed (g), and then volume and gonadosomatic index (%) were calculated. Then, the ovaries were sectioned longitudinally, placed in Bouin’s fixative for 12 h, then transferred to 70% alcohol and processed routinely with paraffin embedding. After processing, 10 µm serial sections were cut, mounted on slides, dried, deparaffinized in xylene, rehydrated in graded ethanol solutions, and 20 equidistant sections were stained with hematoxylin and eosin (Biopack, CABA, Buenos Aires, Argentina) to be evaluated.

Ovarian follicles were divided into five classes on the basis of morphology and number of follicular cells in the widest cross-section, as previously described [18]: primordial (one layer of flattened or flattened cuboidal granulosa cells surrounding the oocyte), primary (one layer of cuboidal granulosa cells surrounding the oocyte) or secondary (oocytes surrounded by two or more cuboidal layers of granulosa cells) antral (small antral space, intact granulosa and a theca cell layers) and atretic (degenerated granulosa cells with apoptotic nuclei, eosinophilic zona pellucida, thickening of the internal theca and follicular fluid containing cellular debris). All procedures were performed by the same operator.

All histological images were obtained using a microscope (Olympus BX50, Tokyo, Japan) through an attached digital RGB video camera (Evolution VF Color, Q Imaging, USA) and digitalized in a 24-bit true color TIFF format and analyzed using the Image Pro Plus v6.0 (Media Cybernetics, Silver Spring, MA, USA). Subsequently, the total number (Nt) of each follicle type was determined according to Gougeon and Chainy (1987): Nt = No × St × ts/So × do [19], where No = number of follicles observed; St = total number of sections; ts = width of the sections (µm); So = sections observed and do = mean diameter of the nucleus of follicle type. Only follicles with a complete visible oocyte nucleolus were recorded. The relative proportion (%) of each follicle type was also calculated. Finally, the presence or absence of corpora lutea was recorded.

### 2.4. Statistical Analysis

Normality of AMH serum values was confirmed by Shapiro–Wilk test. Anti-Müllerian hormone concentrations were grouped as pre-(PRE), under (TREAT) and post (POST)-treatment and compared by repeated measures ANOVA (SPSS 18.0; SPSS, Chicago, IL, USA). The number of each follicle type was compared between sides (right vs. left) by non-paired Student t-test. Day 105 AMH concentrations were correlated with gross parameters by Pearson correlation test. All the variables were expressed as mean ± SEM and the level of significance was set at *p* < 0.05.

## 3. Results

**Figure 1:**
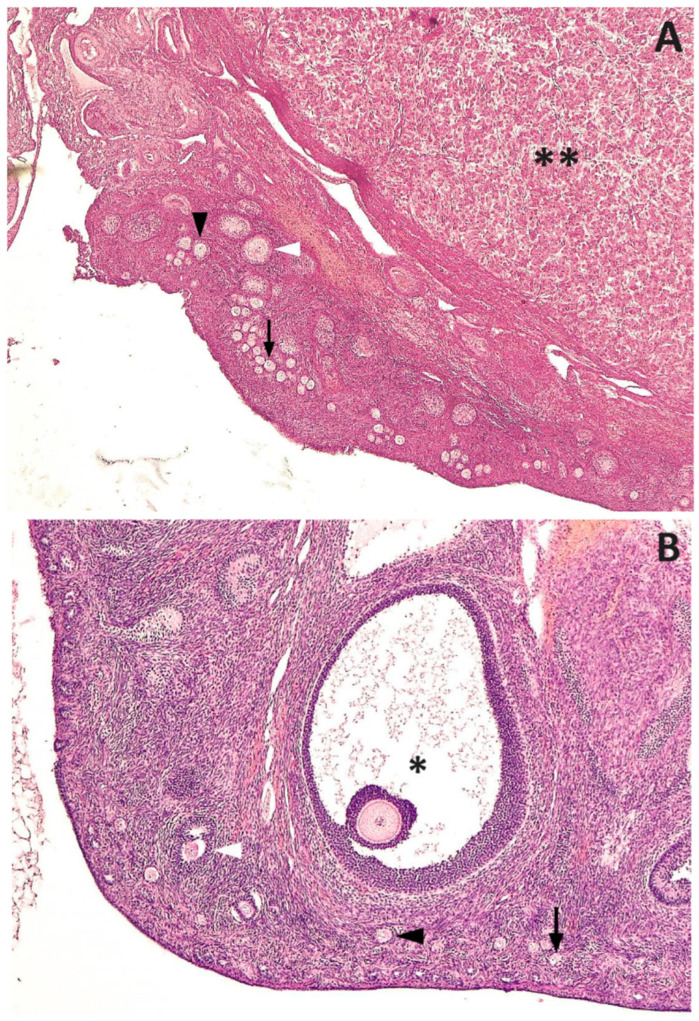
Ovarian tissue of two late anestrous beagle bitches that were serially administered 330 μg/kg SC acyline for 60 days and then spayed 45 days later. (**A**): Corpus luteum (asterisks), primordial follicle (black arrow), primary follicle (black arrow head), secondary follicle (white arrow head). (**B**): Antral follicle (asterisk), primordial follicle (black arrow), primary follicle (black arrow head), secondary follicle (white arrow head). H&E, 40×.

None of the female dogs presented with an estrous cycle during the acyline effect. Only one bitch (Bitch 4) presented as in heat 20 days after treatment, the signs of which ceased at the time of spaying. Histology revealed corpora lutea in this animal (Figure 1A). All the bitches presented primordial, primary and secondary follicles, except one dog (Bitch 3), which also had small antral follicles (Figure 1B). Gross ovarian parameters of these animals are shown in Table 1. The total number of follicles as well as their relative proportion are included in Table 2. No difference was found between the left and right ovaries for any preantral follicle (*p* > 0.1).

**Figure 2:**
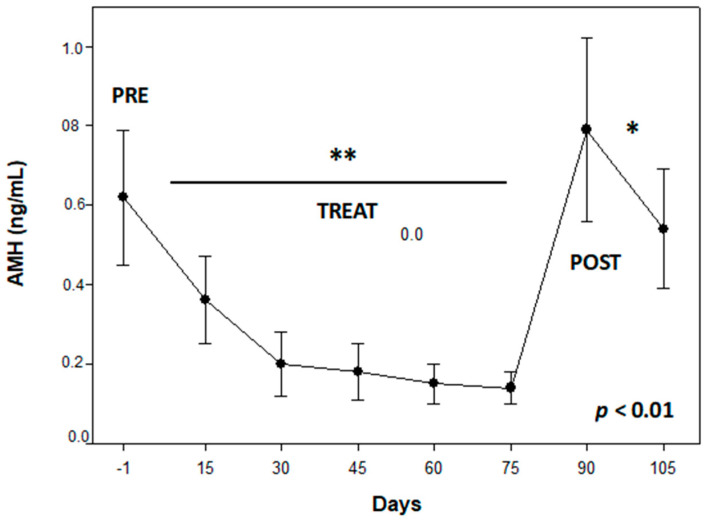
Serum anti-Müllerian hormone (mean ± SEM) concentrations of four late anestrous beagle bitches that were serially administered 330 μg/kg SC acyline for 60 days (TREAT) and then followed up (POST) for 45 days. Different number of asteriks means *p* < 0.01 differences in the study periods.

PRE AMH concentrations were 0.62 ± 0.17 ng/mL. This hormone varied throughout the study period (*p* < 0.01; Figure 2), diminishing to nadir values during TREAT to then rapidly recover in the POST phase (0.2 ± 0.05 vs. 0.67 ± 0.22 ng/mL; *p* < 0.01). The gonadosomatic index was correlated with day 105 AMH concentrations (0.95, *p* < 0.05).

## 4. Discussion

This is the first study to show the effect of a novel GnRH antagonist in anestrous bitches as well as its effect on ovarian follicle population and AMH serum concentrations. The canine estrus cycle is characterized by a long obligate anestrus that can be divided into thirds. Transition from early to late anestrus is characterized by an increase in the amplitude and frequency of GnRH pulses as well as an increase in ovarian responsiveness to gonadotrophins [15]. Thus, two to three months prior to the pre-ovulatory luteinizing hormone surge, small ovarian follicles begin to grow preparing for future ovulation [16].

As expected, in this study, the antagonist suppressed the gonadal axis as no estrus cycle appeared during the treatment effect in any female [5]. One animal (Bitch 4) rapidly cycled after treatment withdrawal and another female (Bitch 3) presented antral follicles 45 days after it, indicating an impending cycle. Bitch 4 also ovulated as evidenced by the presence of the corpora lutea. The other two animals (Bitch 1 and Bitch 2) did not show a histological pattern of advanced follicular development. Grossly, the gonads of these females were in line with previous reports in similar size bitches [20].

Few studies have estimated the total number of preantral follicles in bitches. In line with previous reports, there was a large variation in the numbers of follicles among these females [20,21]. Furthermore, the mean number of follicles found here was similar to the ones previously reported using the same formula [20,21]. Minor differences among studies could be explained by the species variability and operators’ bias. The proportions of the different follicle types were also in line with what has been previously stated for the species [22]. Coincidentally to what has been found before [21], no differences in the number of follicles were found between ovaries, suggesting that in this species, no ovary is more functional. Finally, similarly to what has been shown in hamsters, acyline treatment did not seem to affect follicle count, as in these bitches, the pool appeared normal [23].

GnRH antagonists suppress gonadotrophic secretion, which in turn limits preantral follicle growth [24] and therefore AMH production. Thus, in this canine study, acyline rapidly and reversibly diminished AMH concentrations to nadir values to then recover to initial concentrations quickly after its effect ceased. Conversely, in heifers, acyline (100 µg/kg for 21 days) neither blocked the growth of preantral and small antral follicles nor decreased AMH concentrations [25]. Discrepancies between reports could be due to the known dose effect of GnRH antagonists or to species differences in folliculogenesis [1].

Deslorelin acetate, which has been used as a contraceptive in companion animals, is a GnRH agonist that has a comparable down-regulating effect on the pituitary after an initial short period of stimulation [1]. Cheetahs contracepted with deslorelin presented lower AMH concentrations than untreated females [26]. Domestic queens also showed low AMH concentrations during deslorelin implant treatment. Following implant removal, this hormone rose to pretreatment values in 6 weeks [27]. Coincidentally, in women, combined steroid contraceptive use is associated with a lower mean serum AMH level [28]. If this were proved to be true in canine species, AMH could also be useful to assess the efficacy and duration of traditional hormonal contraceptive protocols.

## 5. Conclusions

The GnRH antagonist acyline rapidly and reversibly prevented the initiation of cycling without affecting the normal follicle count and indirectly diminishing serum AMH concentrations, clearly evidencing the suppression and consequent resumption of ovarian activity. AMH appears to be a functional biomarker to assess the ovarian response to this inhibitory pharmacological protocol.

## Figures and Tables

**Table 1:** Gross ovarian parameters (mean ± SEM) of the four bitches that were serially administered 330 μg/kg SC acyline for 60 days and then spayed 45 days later.

	Mean ± SEM
Body weight (kg)	8.37 ± 0.9
Ovarian volume (cm^2^)	0.5 ± 0.07
Ovarian weight (g)	0.4 ± 0.07
Gonado-somatic index (%)	0.01 ± 0.00

**Table 2:** Ovarian follicles (mean ± SEM) and their relative proportion (%) of the animals of Table 1.

	Mean ± SEM	%
Primordial	80,931.08 ± 23,107.77	84.13
Primary	10,927.42 ± 2536.89	11.36
Secondary	7505.95 ± 3263.16	7.80
Antral	5.55 ± 5.55	0.01
Total	96,200.10 ± 26,125.12	

## Data Availability

The data presented in this study are available on request from the corresponding author.
